# Supplementary material for: Dynamical Modularity in Automata Models of Biochemical Networks
Source: ArXiv. 2023 Mar 29:arXiv:2303.16361v1. Preprint. [Version 1] (PMC10081338)
Supplement: Supplementary file 1 [file NIHPP2303.16361v1-supplement-1.pdf]

arXiv:2303.16361v1 [q-bio.MN] 29 Mar 2023

Supplementary Materials for Dynamical Modularity in Automata Models of Biochemical Networks

March 30, 2023

1 *drosophila* model

The update rules of the single-cell segment polarity network (SPN) are listed here.

Inputs:
$SLP_{t+1}=SLP_t$
$nWG_{t+1}=nWG_t$
$nHH_{t+1}=nHH_t$

Internal nodes:
$wg_{t+1}=(CIA_t \wedge SLP_t \wedge \neg CIR_t) \vee (wg_t \wedge (CIA_t \vee SLP_t) \wedge \neg CIR_t)$
$WG_{t+1}=wg_t$
$en_{t+1}=nWG_t \wedge \neg SLP_t$
$EN_{t+1}=en_t$
$hh_{t+1}=EN_t \wedge \neg CIR_t$
$HH_{t+1}=hh_t$
$ptc_{t+1}=CIA_t \wedge \neg EN_t \wedge \neg CIR_t$
$PTC_{t+1}=ptc_t \vee (PTC_t \wedge \neg nHH_t)$
$PH_{t+1}=PTC_t \wedge nHH_t$
$SMO_{t+1}=\neg PTC_t \vee nHH_t$
$ci_{t+1}=\neg EN_t$
$CI_{t+1}=ci_t$
$CIA_{t+1}=CI_t \wedge (\neg PTC_t \vee nHH_t)$
$CIR_{t+1}=CI_t \wedge PTC_t \wedge \neg nHH_t$

The parasegment SPN (size $n = 60$) extends the single-cell model by including all 14 internal nodes in each of four cells, where WG and HH influence both of the cell's neighbors (periodic boundary conditions assumed), and an additional node, SLP, acts as an input to each cell (see [1] and [2] for a full description).

2 algorithms

Pathway modules can be discovered by a breadth-first search (BFS) on the DCM (*algorithm 1*, see Fig. 1), given the caveat of accounting for the temporal dynamics of state updates. Input to the algorithm is a DCM, a seed set S^0, and a perturbation type (pinning or pulse). The algorithm tracks which s-units and t-units fire in each time step. As with regular BFS units are visited via a first-in first-out (FIFO) queue, but a separate queue is created

for each time step. The time counter is initialized at 0 and increases once all of the units in the associated queue have been visited.

T-units are added to the queue associated with the current time step t if their threshold is met, while s-units are added to the queue associated with time step $t+1$. If the perturbation type is pinning, any given unit is only visited once (as they are afterwards assumed to fire every time step). If the perturbation type is pulse, by contrast, units that previously fired are allowed to be visited again in future time steps.

The algorithm halts when no new s-units or t-units can be added to a queue (logically, no more units can fire) or the set of s-units and t-units that fire at time $t = i$ is equivalent to the set of s-units and t-units that fired at a previous iteration $t = j < i$ (a cycle is reached) [1].

The algorithm returns a hash table keyed by time step, where each value is the set of s-units and t-units that fire in the associated time step. The total set of s-units that fire, S, can then be easily derived by finding the superset of s-units from each time step (and ignoring all t-units).

This algorithm can be extended to the general case where each seed s-unit x is associated with a firing schedule,

$f(x, t) = \{1$ if x is forced to fire at time t,

0 if x is not forced to fire at time $t\}$,

rather than only allowing pinning or pulse perturbation. In this case, the external perturbations dictated by the firing schedule take precedence over any units already in the queue. Additionally, the delay associated with units can be generalized; here all s-units take one time step to fire (i.e., they have delay $dt = 1$); however, s-units can be allowed a higher delay $dt > 1$ such that it takes several (relative) time steps for a signal to reach an s-unit (for example, this may represent cellular processes that operate on a longer time scale than transcription or translation and could be useful for non-synchronous updating schemes [3, 4]).

The set of pathway modules discovered by *algorithm 1* can then be reduced to a set of complex modules (*algorithm 2*, see Fig. 2). Input to the algorithm is a DCM, a maximum seed set size *max_s*, a set of s-units *seeds*, and a perturbation type (pinning or pulse). Algorithm 2 initially finds all pathway modules with seed set size $s = 1$ whose seed is in the set *seeds* using *algorithm 1* and then removes any subsumed modules to create an initial set of complex modules I_1. It then iteratively finds the set of complex modules $I_{s>1}$ with increasing seed set size $s = 2, 3, 4...$*max_s*. For each size s, pathway modules are found for all possible seeds sets, where seeds are obtained from the set *seeds*. This set of potential modules is then reduced to a set of complex modules by removing all modules that are not maximal or that contain non-synergistic submodules. The algorithm halts when the maximum seed set size *max_s* has been reached and returns the set of complex modules found thus far.

The set *seeds* can be any set of s-units of interest (for example, the s-units representing the network inputs). If *seeds*$= \mathcal{S}$, the set of all s-units in the network, then all pathway modules (and therefore all complex modules) will be found; if, however, *seeds*$= \Lambda_1$, then only complex modules with maximal seeds will be found [2]. This is the maximal seed heuristic which finds all core complex modules in the network.

---

[1] This only applies to pulse perturbation where the set of units that fire is reset after each time step; for pinning perturbation, this condition is equivalent to the previous one (i.e., no new units are added to the queue) since all units that fire at time t also fire at time $t+1$.

[2] Λ_1 can be found by running *algorithm 2* with *max_s*$= 1$, *seeds*$= \mathcal{S}$.

All analysis and code for this paper, including *algorithm 1* and *algorithm 2*, was written in Python 2.7 with the assistance of the CANA package for finding Boolean network DCMs [5]. The code is publicly available at https://github.com/tjparmer/dynamical_modularity.

Algorithm 1: Pathway module breadth-first search

---

function thresholded_BFS (*DCM*, S^0, *perturbation_type*):

> time t=0
> *time_steps*={t: queue(S^0)}
> *unfolding*={t: {}}
> *active_step*={S^0}
> *visited*={}
>
> **while** $t \leqslant$max(*time_steps*):
>
> > *visited* $\leftarrow$ *active_step*
> > **if** *active_step* $=$ *unfolding*[$i < t$]:
> > > **return** *unfolding*
> >
> > **if** *perturbation_type*='pulse':
> > > *active_step*={}
> > > **reset** t-unit thresholds
> >
> > **while** *time_steps*[t]:
> > > **pop** first unit x from queue at *time_steps*[t]
> > > *unfolding*[t] $\leftarrow x$
> > > **for each** *DCM* neighbor (s-unit/t-unit) $n \notin$ *active_step*:
> > > > **if** n is not a contradiction **and** the threshold τ of n is met:
> > > > > *dt* = the delay of n
> > > > > *time_steps*[t+*dt*] $\leftarrow n$
> > > > > *active_step* $\leftarrow n$
> >
> > $t = t + 1$
>
> **return** *unfolding*

Figure 1: **Pseudo-code for algorithm 1.** Input to the function is a DCM, a seed set of s-units S^0, and a perturbation type ('pinning' or 'pulse'). The algorithm is similar to breadth-first search (BFS) and explores what part of the DCM is reachable from the seed set, factoring in the perturbation type, relative time steps, logical contradiction, and t-unit thresholds. FIFO queues hashed by relative time step are stored in *time_steps* while sets of visited units hashed by relative time step are stored in *unfolding*. Units (s or t) are only added to the queue associated with the respective time step if they meet the requisite conditions. S-units are given a default threshold $\tau = 1$ and delay $dt = 1$, while t-units have delay $dt = 0$. Units that are visited are added to the sets *visited* and *active_step*. Each t-unit must track the s-units that contribute towards its threshold; with pulse perturbation, unit thresholds are reset after each time step. The algorithm halts when a repeat set of s-units and t-units is found for a given time step or when no new units can be reached based on their logical conditions. The function returns the pathway module $\mathbf{M}_{S^0}$ initiated by the seed set, i.e., the sets of units visited at each relative time step. In the above pseudo-code, {key:value} represents a hash table and {} a set.

Algorithm 2: Complex module discovery

```
function complex_module_search (DCM, max_s, seeds, perturbation_type):

    iteration s=1
    complex modules I={}

    while s ⩽ max_s:
        if s=1:
            new_seeds=seeds
        else:
            new_seeds={S_i^0 ∪ {seed} for i ∈ P, seed ∈ seeds}

        pathway modules P={}
        for seed in new_seeds:
            P ← thresholded_BFS(DCM, seed, perturbation_type)

        for module p ∈ P:
            if p is subsumed by m for any module m ∈ P:
                continue
            else if μ(S_p^0) − μ(S_ps^0) − μ(S_p^0 − S_ps^0) = ∅ for any submodule ps of p:
                continue
            else:
                I ← p
        s = s + 1

    return I
```

Figure 2: **Pseudo-code for algorithm 2.** Input to the function is a DCM, the maximum seed set size *max_s*, a set of s-units *seeds*, and a perturbation type ('pinning' or 'pulse'). At each iteration, new seed sets of size $s = k$ are generated by combining seed sets of current pathway modules of size $s = k - 1$ with the members of *seeds* (size $s = 1$). The pathway modules for these new seed sets are found using algorithm 1; this set of modules is then condensed to a set of complex modules by removing those that are subsumed by other modules or that fail a synergy check. For pinning perturbation, the former condition filters any module p where $S_p \subset S_m$ for any other module $m \in P$, whereas for pulse perturbation the temporal order of when units fire must be checked as well. The function returns the set of complex modules I. In the above pseudo-code, {} represents a set.

3 complex modules and dynamical unfolding

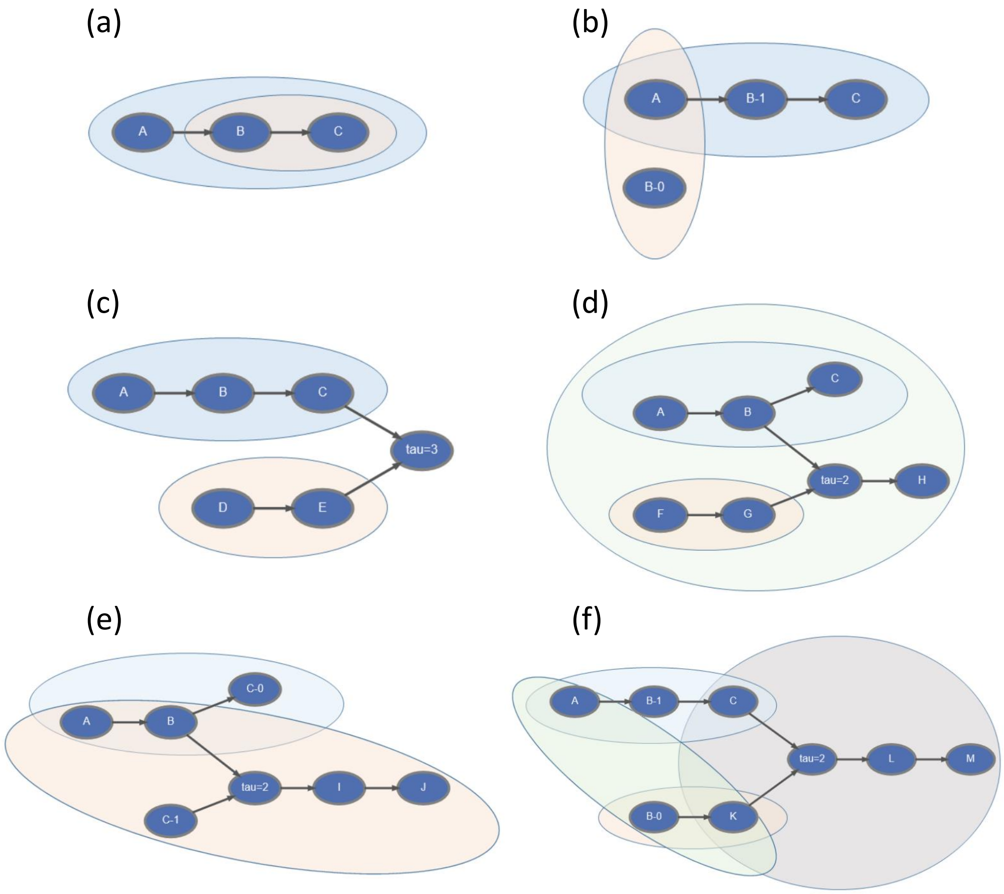

Figure 3: **Example interactions between pathway modules.** (a) Module $\mathbf{M}_A$ subsumes $\mathbf{M}_B$ as the unfolding of $\mathbf{M}_B$ is completely contained in $\mathbf{M}_A$. (b) $\mathbf{M}_{A,B-0}$ logically obstructs $\mathbf{M}_A$ because of the contradiction between B-0 and B-1, which prevents the rest of $\mathbf{M}_A$ from unfolding. (c) $\mathbf{M}_A$ and $\mathbf{M}_D$ are decoupled (dynamically independent); even though they share a threshold, $\mathbf{M}_{A,D}$ does not fire the t-unit and thus cannot cause any downstream effects. (d) $\mathbf{M}_A$ and $\mathbf{M}_F$ are synergistic because $\mathbf{M}_{A,F}$ fires a t-unit that neither module can fire by itself, thereby reaching the node H. (e) There is both synergy and logical obstruction present between $\mathbf{M}_A$ and $\mathbf{M}_{C-1}$, as a logical contradiction with C-1 blocks the complete unfolding of $\mathbf{M}_A$; however, the synergy present also allows $\mathbf{M}_{A,C-1}$ to include I and J. (f) An example of synergy between non-maximal seeds. $\mathbf{M}_C$ is subsumed by $\mathbf{M}_A$ and $\mathbf{M}_K$ is subsumed by $\mathbf{M}_{B-0}$. Because of the contradiction of B-0 and B-1, $|S_{A,B-0}| = 3$ (shown in green). However, if C and K are used as seeds, then a larger module is seen which includes the extra nodes L and M, $|S_{C,K}| = 4$ (shown in brown). This synergy is found in two complex modules, $|S_{C,B-0}| = 5$ and $|S_{A,K}| = 6$; however, neither of these are core complex modules because $\mathbf{M}_C$ and $\mathbf{M}_K$ are not maximal.

$\mathbf{M}_i$	dynamical unfolding	$\lvert S_i \rvert$
$\mathbf{M}_{\text{en-1}}$ (Σ_2)	EN-1, *ci-0*, *ptc-0*, CI-0, CIR-0, CIA-0, *hh-1*, HH-1	9
$\mathbf{M}_{\text{nWG-0}}$ (Σ_1)	*en-0*, EN-0, *ci-1*, *hh-0*, CI-1, HH-0	7
$\mathbf{M}_{\text{SLP-1}}$ (Σ_1)	*en-0*, EN-0, *ci-1*, *hh-0*, CI-1, HH-0	7
$\mathbf{M}_{\text{CIR-1}}$	*hh-0*, *wg-0*, *ptc-0*, HH-0, WG-0	6
$\mathbf{M}_{\text{PTC-0}}$	SMO-1, PH-0, CIR-0	4
$\mathbf{M}_{\text{HH-1}}$	SMO-1, CIR-0	3
$\mathbf{M}_{\text{HH-0}}$	PH-0	2
$\mathbf{M}_{\text{wg-1}}$	WG-1	2
$\mathbf{M}_{\text{ptc-1}}$	PTC-1	2
$\mathbf{M}_{\text{nWG-1}}$	–	1
$\mathbf{M}_{\text{SLP-0}}$	–	1
$\mathbf{M}_{\text{PH-1}}$	–	1
$\mathbf{M}_{\text{SMO-0}}$	–	1
$\mathbf{M}_{\text{CIA-1}}$	–	1
$\mathbf{M}_{\text{SLP-1,nHH-1}}$ (Σ_3)	SMO-1, CIR-0, *en-0*, EN-0, *ci-1*, *hh-0*, CI-1, HH-0, CIA-1, *wg-1*, *ptc-1*, WG-1, PTC-1, PH-1	16
$\mathbf{M}_{\text{SLP-1,PTC-0}}$ (Σ_5)	SMO-1, PH-0, CIR-0, *en-0*, EN-0, *ci-1*, *hh-0*, CI-1, HH-0, CIA-1, *wg-1*, *ptc-1*, WG-1	15
$\mathbf{M}_{\text{nWG-0,nHH-1}}$ (Σ_3)	SMO-1, CIR-0, *en-0*, EN-0, *ci-1*, *hh-0*, CI-1, HH-0, CIA-1, *ptc-1*, PTC-1, PH-1	14
$\mathbf{M}_{\text{nWG-0,PTC-0}}$ (Σ_5)	SMO-1, PH-0, CIR-0, *en-0*, EN-0, *ci-1*, *hh-0*, CI-1, HH-0, CIA-1, *ptc-1*	13
$\mathbf{M}_{\text{nHH-1,en-1}}$ (Σ_4)	SMO-1, CIR-0, EN-1, *hh-1*, *ci-0*, *ptc-0*, HH-1, CI-0, PTC-0, PH-0, CIA-0	13
$\mathbf{M}_{\text{SLP-0,nWG-1}}$ (Σ_2)	*en-1*, EN-1, *ci-0*, *ptc-0*, CI-0, CIR-0, CIA-0, *hh-1*, *wg-0*, HH-1, WG-0	13
$\mathbf{M}_{\text{nHH-1,CIR-1}}$	SMO-1, *hh-0*, *wg-0*, *ptc-0*, HH-0, WG-0, PTC-0, PH-0	10
$\mathbf{M}_{\text{nHH-0,ptc-1}}$	PH-0, PTC-1, SMO-0, CIA-0	6
$\mathbf{M}_{\text{SLP-0,nWG-1,nHH-1}}$ (Σ_4)	SMO-1, CIR-0, *en-1*, EN-1, *hh-1*, *ci-0*, *ptc-0*, HH-1, CI-0, PTC-0, PH-0, CIA-0, *wg-0*, WG-0	17
$\mathbf{M}_{\text{SLP-1,nHH-1,CIR-1}}$	SMO-1, *hh-0*, *wg-0*, *ptc-0*, *en-0*, HH-0, WG-0, PTC-0, EN-0, *ci-1*, PH-0, CI-1, CIA-1	16
$\mathbf{M}_{\text{nWG-0,nHH-1,CIR-1}}$	SMO-1, *hh-0*, *wg-0*, *ptc-0*, *en-0*, HH-0, WG-0, PTC-0, EN-0, *ci-1*, PH-0, CI-1, CIA-1	16
$\mathbf{M}_{\text{SLP-1,nHH-0,ptc-1}}$	PH-0, PTC-1, *en-0*, SMO-0, CIA-0, EN-0, *ci-1*, *hh-0*, CI-1, HH-0, CIR-1, *wg-0*, WG-0	16
$\mathbf{M}_{\text{nWG-0,nHH-0,ptc-1}}$	PH-0, PTC-1, *en-0*, SMO-0, CIA-0, EN-0, *ci-1*, *hh-0*, CI-1, HH-0, CIR-1, *wg-0*, WG-0	16
$\mathbf{M}_{\text{SLP-0,nHH-0,ptc-1}}$	PH-0, PTC-1, SMO-0, CIA-0, *wg-0*, WG-0	9

Table 1: **List of the core complex modules in the *drosophila* single-cell SPN with pinning perturbation.** Modules are grouped by seed set size and ordered by resulting size $\lvert S_i \rvert$. The module labels are colored to correspond to Figure 5 in the main text, as well as supplementary figures 4, 5, and 6; membership in a set of related modules Σ is also indicated in parentheses. The s-units are listed in the temporal order that they are visited within the dynamical unfolding process.

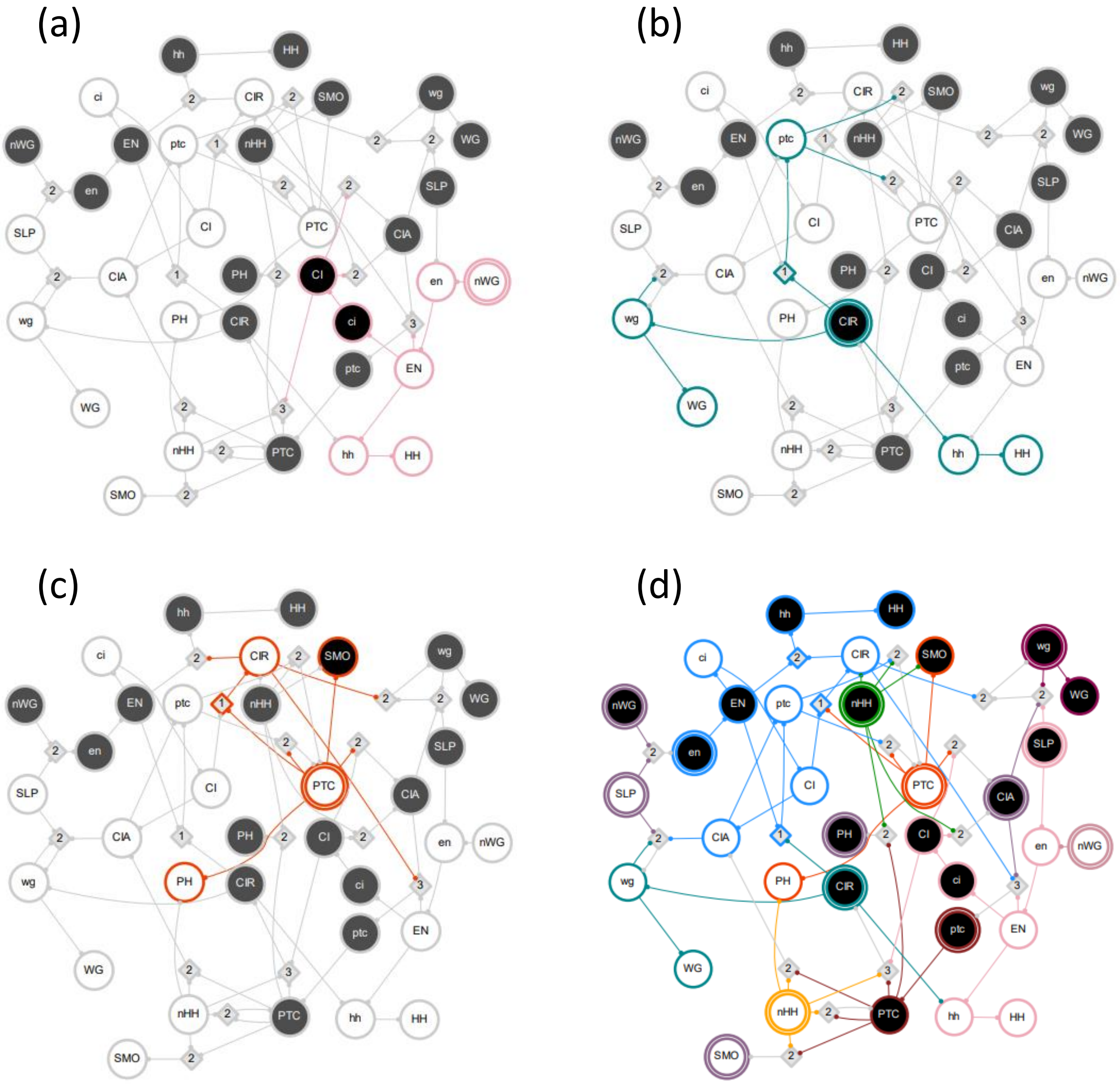

Figure 4: **Complex modules in the *drosophila* single-cell SPN, seed set size $s = 1$.** Select complex modules (indicated by color) are shown: module $\mathbf{M}_{nWG-0}$ (a), module $\mathbf{M}_{CIR-1}$ (b), module $\mathbf{M}_{PTC-0}$ (c), and all 14 complex modules with seed set size $s = 1$ (d). These modules dynamically unfold from their seed set of size $s = 1$ (highlighted with a double edge) to include the s and t-units that are guaranteed to fire given pinning perturbation. In panel d, the 14 modules indicated compose Λ_1 and cover the DCM. S-units and t-units are colored by the largest module that they are a part of; modules that only contain the seed ($|S_i| = 1$) are in purple. Note that several t-units have been removed or modified in the figure for clarity of the transition function.

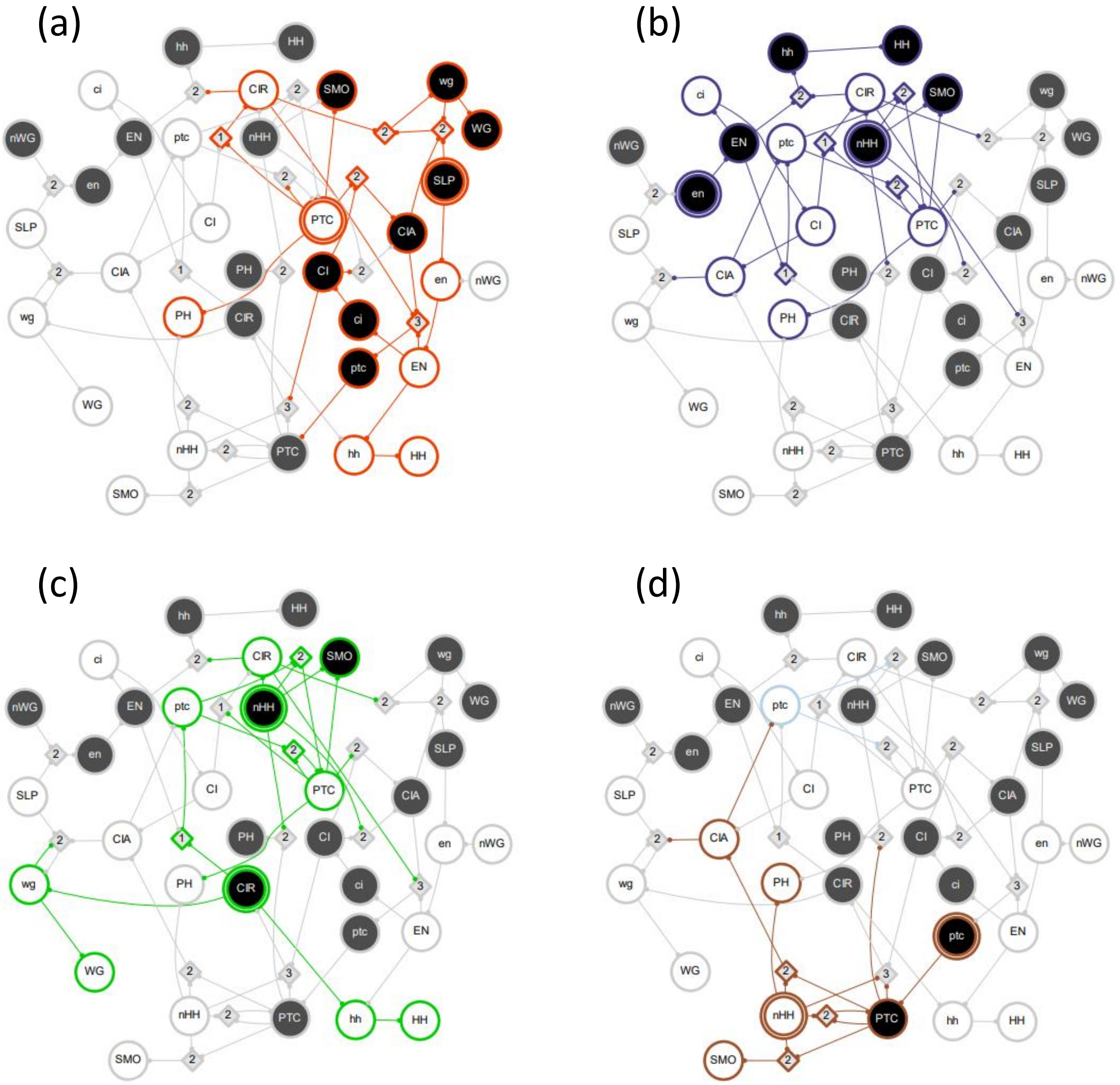

Figure 5: **Complex modules in the *drosophila* single-cell SPN, seed set size** $s = 2$**.** Select complex modules (indicated by color) are shown: module $\mathbf{M}_{SLP-1,PTC-0}$ (a), module $\mathbf{M}_{nHH-1,en-1}$ (b), module $\mathbf{M}_{nHH-1,CIR-1}$ (c), and module $\mathbf{M}_{nHH-0,ptc-1}$ (d). These modules dynamically unfold from their seed set of size $s = 2$ (highlighted with a double edge) to include the s and t-units that are guaranteed to fire given pinning perturbation. In panel d, note that s-unit *ptc-0* does not fire with pinning perturbation due to contradiction with *ptc-1*. Note that several t-units have been removed or modified in the figure for clarity of the transition function.

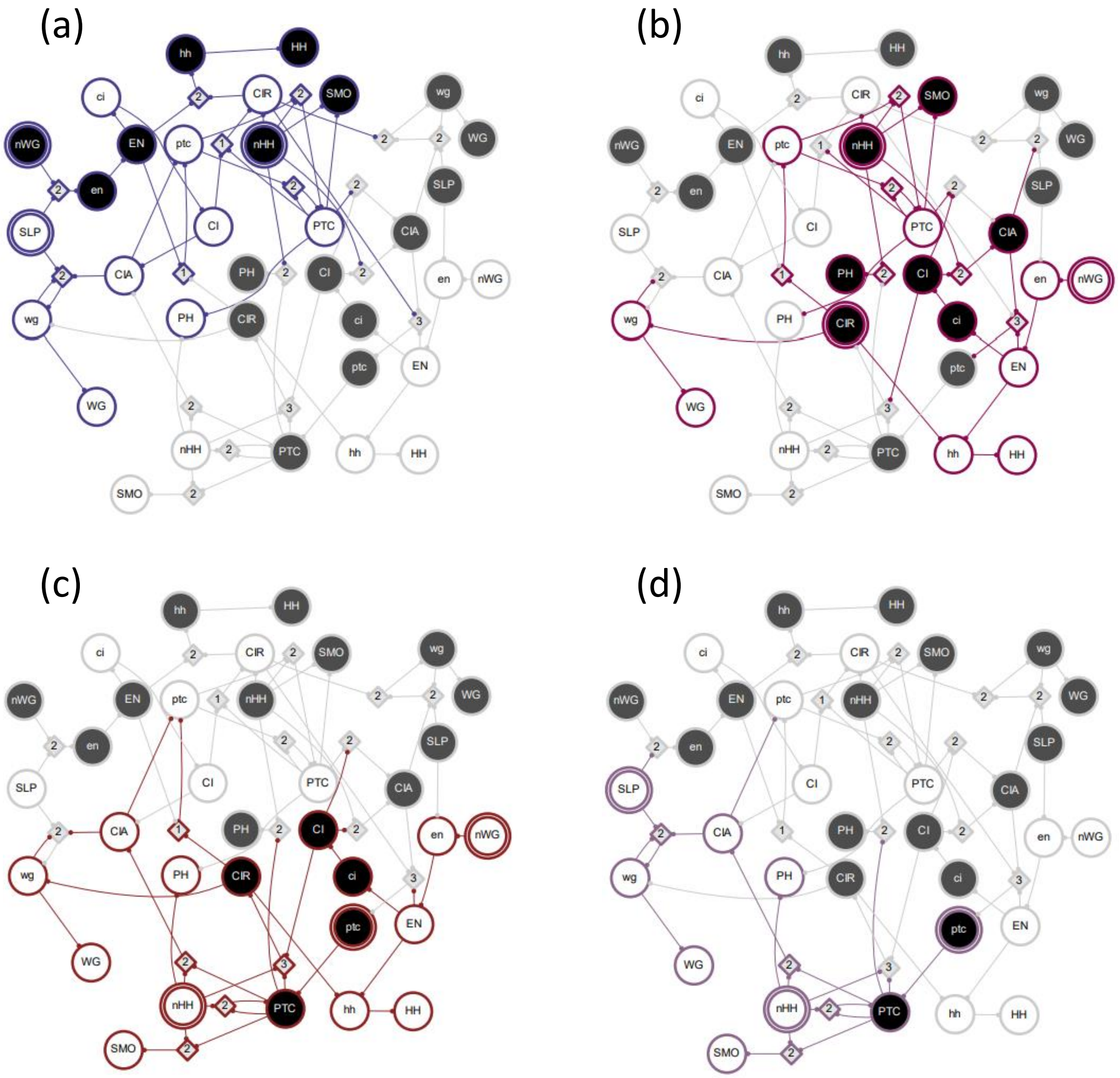

Figure 6: **Complex modules in the *drosophila* single-cell SPN, seed set size** $s = 3$**.** Select complex modules (indicated by color) are shown: module $\mathbf{M}_{SLP-0,nWG-1,nHH-1}$ (a), module $\mathbf{M}_{nWG-0,nHH-1,CIR-1}$ (b), module $\mathbf{M}_{nWG-0,nHH-0,ptc-1}$ (c), and module $\mathbf{M}_{SLP-0,nHH-0,ptc-1}$ (d). These modules dynamically unfold from their seed set of size $s = 3$ (highlighted with a double edge) to include the s and t-units that are guaranteed to fire given pinning perturbation. In panel a, the indicated module resolves all 17 variable states and results in one of the network's 10 attractors. In panel b, the indicated module is identical to $\mathbf{M}_{SLP-1,nHH-1,CIR-1}$ but differs only in the seed. In panel c, the indicated module is identical to $\mathbf{M}_{SLP-1,nHH-0,ptc-1}$ but differs only in the seed. Note that several t-units have been removed or modified in the figure for clarity of the transition function.

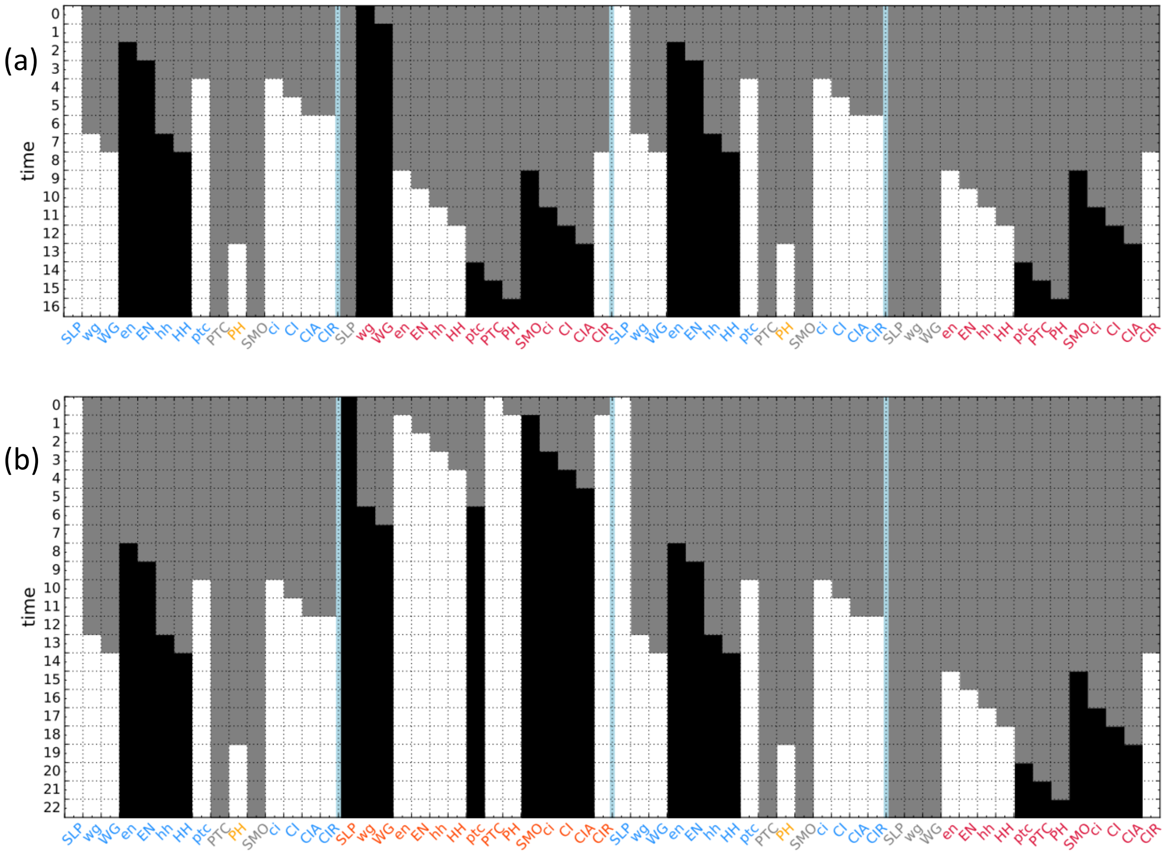

Figure 7: **Dynamical unfolding in the *drosophila* parasegment SPN.** Cell boundaries are separated by a blue line; white indicates that a node is OFF in that time step, black indicates that a node is ON, and grey indicates that the node's state is unknown. The color of the node label indicates the associated complex module. (a) The dynamical unfolding of $\text{SLP}_1 = \text{SLP}_3 = 0$, $wg_2 = 1$. The presence of WG-1 in cell 2 allows $\mathbf{M}_{\text{SLP}-0,n\text{WG}-1}$ (labeled as blue) to unfold in cells 1 and 3 by acting as an additional input to SLP-0. These modules turn off WG and turn on HH in cells 1 and 3, which initiates $\mathbf{M}_{n\text{WG}-0,n\text{HH}-1}$ in cells 2 and 4 (labeled as red). Finally $\mathbf{M}_{n\text{WG}-0,n\text{HH}-1}$ in cells 2 and 4 turns off HH which initiates the (small) $\mathbf{M}_{n\text{HH}-0}$ module in cells 1 and 3 (labeled as yellow). This resolves almost the entire network (52 variables) with only three inputs. (b) The dynamical unfolding of $\text{SLP}_1 = \text{SLP}_3 = \text{PTC}_2 = 0$, $\text{SLP}_2 = 1$ is almost identical, except instead of *wg*-1, SLP-1 and PTC-0 are pinned in cell 2. $\mathbf{M}_{\text{SLP}-1,\text{PTC}-0}$ (labeled as orange) similarly turns on WG, initiating the $\mathbf{M}_{\text{SLP}-0,n\text{WG}-1}$ module in cells 1 and 3. However, the time differences are more pronounced. The unfolding can be seen in three distinct epochs where first $\mathbf{M}_{\text{SLP}-1,\text{PTC}-0}$ must unfold in cell 2, then the $\mathbf{M}_{\text{SLP}-0,n\text{WG}-1}$ modules are initiated in cells 1 and 3, and then $\mathbf{M}_{n\text{WG}-0,n\text{HH}-1}$ is initiated in cell 4. This demonstrates how important timing is in the dynamic interplay between cells.

$\lvert S_i^0 \rvert$	S_i^0	$\lvert S_i \rvert$	associated modules
$s = 1$	*en*-1_i	13	$\mathbf{M}_2/\mathbf{M}_3$
$s = 2$	(*en*-1_i, *en*-1_{i+1}),<br>(CIR-1_i, CIR-1_{i+2})	28	$\mathbf{M}_8/\mathbf{M}_3$,<br>$\mathbf{M}_5/\mathbf{M}_1/\mathbf{M}_4$
$s = 3$	{ (SLP-0_i, SLP-0_{i+2}, *wg*-1_{i+1}) } = X	52	$\mathbf{M}_7/\mathbf{M}_6/\mathbf{M}_4$
$s = 4$	{ (X + *en*-1_{i+1}),<br>(X + *en*-1_{i+3}) } = Y	56	$\mathbf{M}_8/\mathbf{M}_{10}/\mathbf{M}_6$,<br>$\mathbf{M}_8/\mathbf{M}_{10}/\mathbf{M}_6$
$s = 5$	{ (SLP-1_i, SLP-1_{i+1}, SLP-0_{i+2}, *wg*-1_{i+3}, *en*-1_{i+3}),<br>(SLP-1_i, SLP-1_{i+1}, SLP-0_{i+3}, *wg*-1_{i+2}, *en*-1_{i+2}),<br>(Y + SLP_1_{i+3}),<br>(Y + SLP_0_{i+3}),<br>(SLP-0_i, SLP-0_{i+1}, SLP-1_{i+2}, *wg*-1_{i+3}, *en*-1_{i+3}),<br>(SLP-0_i, SLP-0_{i+1}, SLP-1_{i+3}, *wg*-1_{i+2}, *en*-1_{i+2}) } = Z	59	$\mathbf{M}_8/\mathbf{M}_{10}/\mathbf{M}_6$<br>...
$s = 6$	(Z + SLP-0_j),<br>(Z + SLP-1_j)<br>(*en*-1_i, PTC-0_{i+2}, SLP-0_i, SLP-0_{i+1}, SLP-1_{i+2}, SLP-1_{i+3})<br>(*en*-1_i, PTC-0_{i+2}, SLP-0_i, SLP-1_{i+1}, SLP-1_{i+2}, SLP-0_{i+3})<br>(*en*-1_i, PTC-0_{i+2}, SLP-0_i, SLP-0_{i+1}, SLP-1_{i+2}, SLP-0_{i+3})	60	$\mathbf{M}_8/\mathbf{M}_{10}/\mathbf{M}_6$<br>...<br>$\mathbf{M}_{10}/\mathbf{M}_6/\mathbf{M}_9$

Table 2: **Maximal modules in the *drosophila* parasegment SPN.** The seed set S_i^0 and size $\lvert S_i \rvert$ of the maximal pathway module per seed set size $s = \lvert S_i^0 \rvert$ are shown, along with the associated intracellular complex modules found within the maximal module. Here, $\mathbf{M}_1$ indicates $\mathbf{M}_{nWG-0}$; $\mathbf{M}_2$ indicates $\mathbf{M}_{en-1}$; $\mathbf{M}_3$ indicates $\mathbf{M}_{nHH-1}$; $\mathbf{M}_4$ indicates $\mathbf{M}_{nHH-0}$; $\mathbf{M}_5$ indicates $\mathbf{M}_{CIR-1}$; $\mathbf{M}_6$ indicates $\mathbf{M}_{SLP-1,nHH-1}$; $\mathbf{M}_7$ indicates $\mathbf{M}_{SLP-0,nWG-1}$; $\mathbf{M}_8$ indicates $\mathbf{M}_{nHH-1,en-1}$; $\mathbf{M}_9$ indicates $\mathbf{M}_{PTC-0}$; finally, $\mathbf{M}_{10}$ indicates $\mathbf{M}_{SLP-0,nWG-1,nHH-1}$. For $s = 5$, all maximal pathway modules have the same associated intracellular modules; for $s = 6$, the first two groupings of seed sets have associated modules $\mathbf{M}_8$, $\mathbf{M}_{10}$, and $\mathbf{M}_6$, while the latter three groupings have associated modules $\mathbf{M}_{10}$, $\mathbf{M}_6$, and $\mathbf{M}_9$. The subscript on the s-unit labels denotes the cell of the parasegment where i and j are arbitrary cells chosen such that there is no logical contradiction between variable states and cellular addition uses modular arithmetic. As maximal modules with larger seed sets tend to build on the same set of modules with smaller seed sets, particular seed sets are condensed to variables for readability. Given the periodic boundaries of the parasegment and the similarity of complex modules, a significant degree of redundancy in regards to cellular indices can be seen in the maximal module sets. A seed set of size $s \geqslant 6$ is needed to resolve every variable in the network.

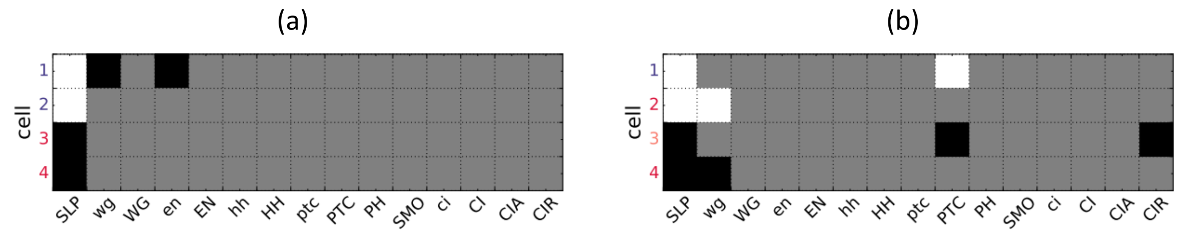

Figure 8: **Minimal schemata for attractor control in the *drosophila* parasegment SPN.** (a) One of the minimal schemata found to reach an attractor, representing the minimal seeds sufficient to drive the dynamics to that attractor with pinning control. Each row represents a cell in the *drosophila* parasegment and each column the state of the respective node: black represents ON, white represents OFF, and grey represents a wildcard value (i.e., the node state is redundant for convergence to the attractor under pinning perturbation of the seeds). The color of the row label represents the main module group active in that cell during unfolding (pink represents Σ_1, red represents Σ_3, and violet represents Σ_4, see Table 1). (b) Same as panel a, but for the minimal schema sufficient to reach the wildtype attractor. The minimal configurations found are much smaller than the previous estimates without pinning perturbation found in [2].

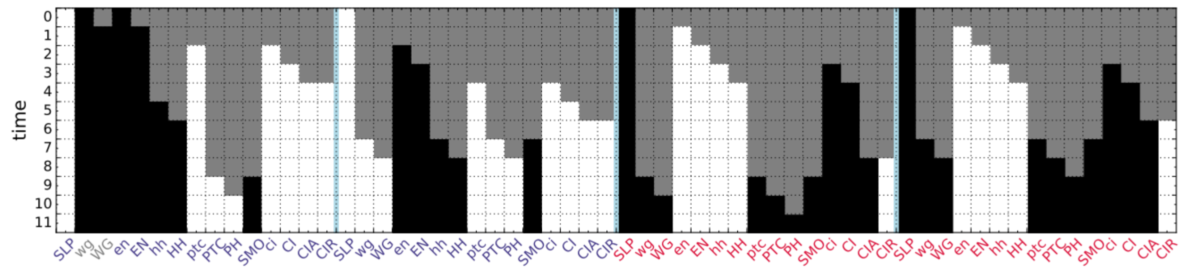

Figure 9: **Dynamical unfolding of a minimal seed set to reach an attractor in the *drosophila* parasegment SPN.** Cell boundaries are separated by a blue line; white indicates that a node is OFF in that time step, black indicates that a node is ON, and grey indicates that the node's state is unknown. The color of the node label indicates the associated complex module. All four SLP inputs are pinned; additionally, *wg* and *en* are ON in the first cell. $\mathbf{M}_{en-1}$ unfolds in cell 1, and *wg*-1 initiates $\mathbf{M}_{SLP-0,nWG-1}$ in cell 2 by acting as an input along with SLP-0. This turns on HH in both cells which extends the unfolding in each cell via additional synergy (modules $\mathbf{M}_{en-1,nHH-1}$ and $\mathbf{M}_{SLP-0,nWG-1,nHH-1}$, labeled as violet). It also extends the unfolding of $\mathbf{M}_{SLP-1}$ in cells 3 and 4 (module $\mathbf{M}_{SLP-1,nHH-1}$, labeled as red). This final configuration is in the basin of attraction of the broad stripes attractor [1], and the network will reach this attractor once the seeds *wg* and *en* in cell 1 are no longer pinned.

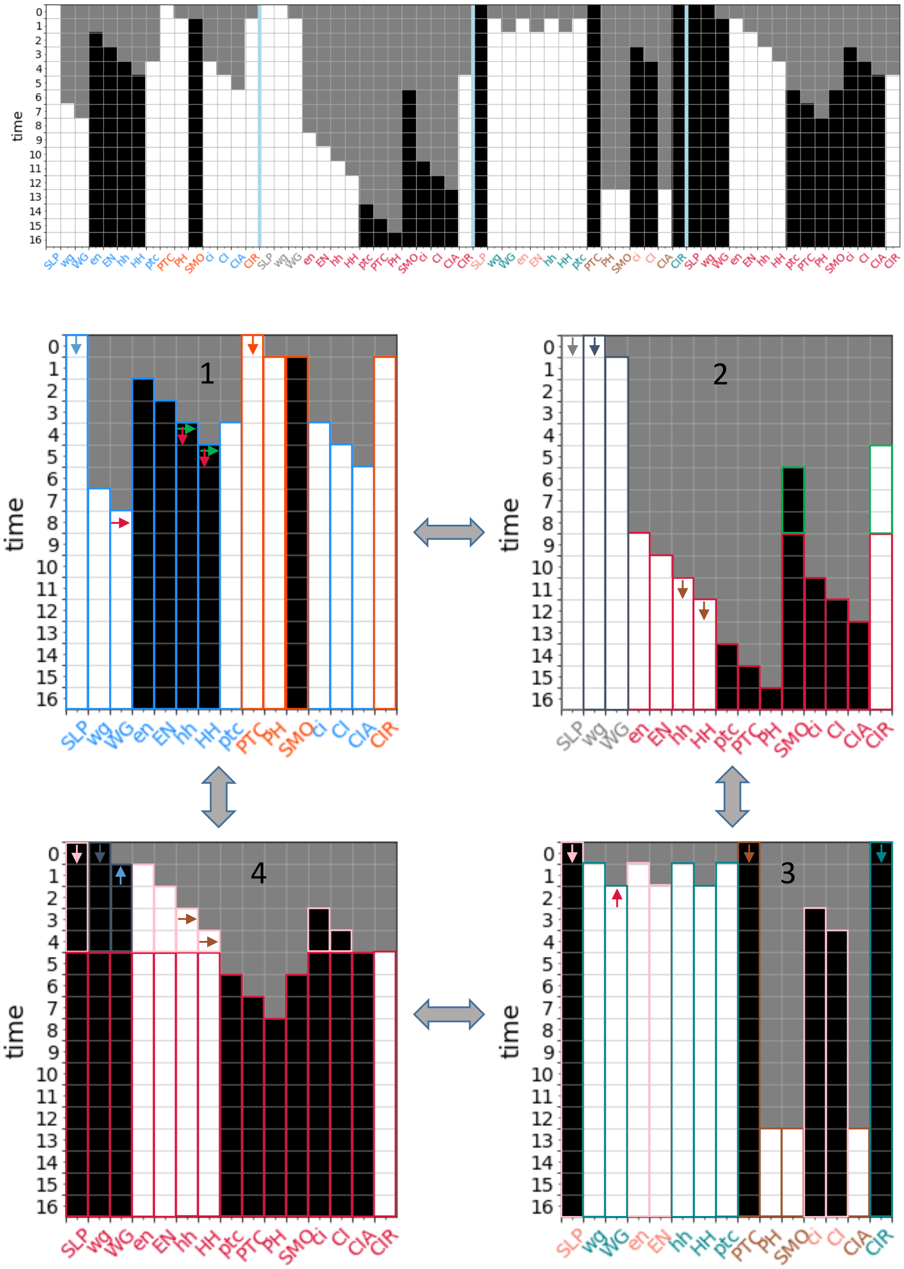

Figure 10: **Dynamical unfolding of the wildtype attractor in the *drosophila* parasegment SPN.** This figure is the same as Fig. 6 in the main text except that the full unfolding of the minimal seed set to the wildtype attractor is shown in terms of intracellular complex modules. $\mathbf{M}_{SLP-0}$ is present in cell 1 based on initial conditions (blue arrow). The influence of WG-1 in cell 4 (blue arrow) initiates $\mathbf{M}_{SLP-0,nWG-1}$ in cell 1 (shown by blue borders). This module turns on *hh* and HH which initiates $\mathbf{M}_{nHH-1}$ in cell 2 (shown in green) and then turns off WG in cell 1 which, together with WG-0 in cell 3 (indicated by red arrows), initiates $\mathbf{M}_{nWG-0}$ in cell 2; the synergy between $\mathbf{M}_{nHH-1}$ and $\mathbf{M}_{nWG-0}$ results in $\mathbf{M}_{nHH-1,nWG-0}$ in cell 2 (which subsumes both modules and is shown by red borders). Based on initial conditions, $\mathbf{M}_{PTC-0}$ is also present in cell 1, $\mathbf{M}_{SLP-0}$ and $\mathbf{M}_{wg-0}$ are present in cell 2, $\mathbf{M}_{SLP-1}$ and $\mathbf{M}_{CIR-1}$ are present in cell 3, and $\mathbf{M}_{SLP-1}$ and $\mathbf{M}_{wg-1}$ are present in cell 4. $\mathbf{M}_{SLP-1}$ in cell 4 (shown by pink borders) turns off *hh* and HH which, together with *hh*-0 and HH-0 in cell 2 (indicated by brown arrows), initiates $\mathbf{M}_{nHH-0}$ in cell 3, which is subsumed by $\mathbf{M}_{nHH-0,PTC-1}$ (shown by brown borders). Finally, the presence of *hh*-1 and HH-1 in cell 1 initiates $\mathbf{M}_{nHH-1}$ in cell 4 , which is subsumed by $\mathbf{M}_{SLP-1,nHH-1}$ (shown in red).

4 *drosophila* OBSERVABILITY AND CONTROL

The single-cell SPN is small enough that the attractors of the network can be fully enumerated. Analysis of the dynamical activity within each of the 10 attractors corresponds well with the analysis of complex modules, as each attractor can be associated with one or more modules (Table 3). The Σ_4 module $\mathbf{M}_{SLP-0,nWG-1,nHH-1}$ is seen in only one attractor as it requires an AND condition between all three inputs, resulting in the driver set (SLP=0, nWG=1, nHH=1). The Σ_3 modules, $\mathbf{M}_{SLP-1,nHH-1}$ and $\mathbf{M}_{nWG-0,nHH-1}$, are seen in four attractors: the former module resolves every variable except for the input nWG and therefore leads to two attractors depending on the state of this input with driver sets (SLP=1, nWG=1, nHH=1) and (SLP=1, nWG=0, nHH=1); the latter module resolves every variable except for SLP, *wg*, and WG and can lead to three attractors, with driver sets (SLP=1, nWG=0, nHH=1) (seen above), (SLP=0, nWG=0, nHH=1, *wg*=0), and (SLP=0, nWG=0, nHH=1, *wg*=1). The modules $\mathbf{M}_{SLP-1,nHH-0,PTC-1}$ and $\mathbf{M}_{nWG-0,nHH-0,PTC-1}$ are identical except for their seeds and lead to three attractors, as both modules resolve every variable state except for the missing input (which my be in either state) [3]. The driver sets for the three attractors are therefore (SLP=1, nWG=0, nHH=0, PTC=1), (SLP=1, nWG=1, nHH=0, PTC=1), and (SLP=0, nWG=0, nHH=0, PTC=1). The final two attractors require $\mathbf{M}_{SLP-0,nWG-1}$ in combination with other modules; one requires $\mathbf{M}_{nHH-0}$ and $\mathbf{M}_{PTC-0}$, while the other requires $\mathbf{M}_{nHH-0,PTC-1}$ (note that there is synergy between nHH-0 and PTC-1 and that this module is also a submodule of $\mathbf{M}_{SLP-1,nHH-0,PTC-1}$ and $\mathbf{M}_{nWG-0,nHH-0,PTC-1}$, meaning that it appears in four attractors). The driver sets for these two attractors are therefore (SLP=0, nWG=1, nHH=0, PTC=0) and (SLP=0, nWG=1, nHH=0, PTC=1). Interestingly, the superset of the nodes associated with the minimal driver sets found via this methodology are the same driver nodes predicted by feedback vertex set theory (inputs SLP, nWG, nHH and internal nodes PTC and *wg*).

The characterization of dynamical modules here highlights the difference between the driver node set with pinning and pulse perturbation control. If a driver node's state must be constant, as with pinning perturbations, PTC and *wg* are needed to fully control the network. However, if one is allowed to flip the states of the input variables, then no internal nodes need to be pinned. For example, to reach the attractor that requires $\mathbf{M}_{SLP-0,nWG-1}$, $\mathbf{M}_{nHH-0}$, and $\mathbf{M}_{PTC-0}$, the module $\mathbf{M}_{SLP-0,nWG-1,nHH-1}$ can be used which turns off PTC (and maintains PTC in the OFF state), and nHH can then be switched OFF to reach the attractor. Furthermore, modules $\mathbf{M}_{SLP-1,nHH-1}$ and $\mathbf{M}_{nWG-0,nHH-1}$ turn off *ptc*; if the state of nHH is then flipped, the module $\mathbf{M}_{nHH-0,PTC-1}$ will unfold, as is seen in four attractors. In general, perturbing nHH along with the other input nodes replaces the need to control PTC as nHH is an input to PTC. Perturbing the internal node *wg*, meanwhile, allows to turn on or off *wg* and WG, when SLP=0, nWG=0, and nHH=1. However, SLP can also be turned ON after $\mathbf{M}_{nWG-0,nHH-1}$ unfolds to turn on *wg* and WG and then turned OFF. Alternatively, SLP can be turned OFF while nWG and nHH are ON to initiate $\mathbf{M}_{SLP-0,nWG-1,nHH-1}$, which turns off *wg* and WG; then nWG can be flipped to initiate $\mathbf{M}_{nWG-0,nHH-1}$. Therefore, perturbing SLP and nWG replaces the need to control *wg*. In a sense, pinning the internal nodes then is just a shortcut to replace the need

[3] These are complex modules but not core complex modules as PTC is not a maximal seed (because $\mathbf{M}_{ptc-1}$ subsumes $\mathbf{M}_{PTC-1}$).

for input node perturbations. This highlights the usefulness of complex modules in developing a qualitative understanding of control in a system.

Perhaps unsurprisingly, the same intracellular modules that lead to attractors in the single-cell SPN lead to attractors in the parasegment SPN, when considering six biologically-relevant attractors from [1]. In particular, $\mathbf{M}_{SLP-0,nWG-1}$, $\mathbf{M}_{nWG-0,nHH-1}$, $\mathbf{M}_{SLP-1,nHH-1}$, and $\mathbf{M}_{SLP-1,nHH-0,PTC-1}$ each play a significant role, appearing in at least four of the attractors (see Table 4).

The logical inferences made during the dynamical unfolding process are also useful in the problem of observability. If a network has reached a fixed point, then each variable state is unchanging (i.e., pinned); thus, observing a set of sensor nodes with corresponding s-units S_i^0 guarantees that the module $\mathbf{M}_{S_i^0}$ has fully unfolded and all the variable states contained in S_i are present in the current state of the network. Observing only a small subset of such states (2-3 in the case of the *drosophila* single-cell SPN, equivalent to less than 18% of the network size) is sufficient to figure out which attractor the network is in. The size of this minimal observability set is naturally upper-bounded by the size of the minimal driver set. For the *drosophila* parasegment SPN, the minimal observability sets require only two s-units (3% of the network size) when considering only the six biologically-relevant attractors in Table 4.

Attractor	Minimal observability sets	Minimal control set	Associated modules
11100001111111010	{ (nHH-1, SLP-1, nWG-0), (SLP-1, *ptc-1*, nWG-0), (*wg-1*, SLP-1, nWG-0), (SLP-1, nWG-0, WG-1), (CIR-0, SLP-1, nWG-0), (SLP-1, nWG-0, CIA-1), (SLP-1, nWG-0, PH-1), (SLP-1, SMO-1, nWG-0) }	(nHH-1, SLP-1, nWG-0)	$\mathbf{M_6}$,$\mathbf{M_{11}}$
01100001111111010	{(*wg*-1, SLP-0), (SLP-0, WG-1)}	(nHH-1, nWG-0, *wg-1*, SLP-0)	$\mathbf{M_{11}}$,$\mathbf{M_{wg\text{-}1}}$,$\mathbf{M_{SLP\text{-}0}}$
11100001111111011	{ (nWG-1, *wg-1*), (*ptc-1*, nWG-1), (nWG-1, PH-1), (nWG-1, CIA-1), (nWG-1, WG-1) }	(nHH-1, nWG-1, SLP-1)	$\mathbf{M_6}$,$\mathbf{M_{nWG\text{-}1}}$
00011110001000011	{ (nHH-1, PTC-0), (nHH-1, *en-1*), (EN-1, nHH-1), (nHH-1, *ci-0*), (CI-0, nHH-1), (nHH-1, CIA-0), (nHH-1, *ptc-0*), (nHH-1, *hh-1*), (HH-1, nHH-1), (nHH-1, PH-0) }	(nHH-1, nWG-1, SLP-0)	$\mathbf{M_{10}}$
10000000100110101	{(CIR-1, nWG-1)}	(SLP-1, nHH-0, PTC-1, nWG-1)	$\mathbf{M_{13}}$,$\mathbf{M_{nWG\text{-}1}}$
00011110001000001	{(nHH-0, PTC-0), (SMO-1, nHH-0)}	(nWG-1, PTC-0, nHH-0, SLP-0)	$\mathbf{M_7}$, $\mathbf{M_9}$, $\mathbf{M_4}$
00000001111111010	{ (*wg-0*, *ptc-1*), (*ptc-1*, WG-0), (*wg-0*, PH-1), (*wg-0*, CIA-1), (WG-0, PH-1), (WG-0, CIA-1) }	(nWG-0, nHH-1, SLP-0, *wg-0*)	$\mathbf{M_{11}}$,$\mathbf{M_{wg\text{-}0}}$,$\mathbf{M_{SLP\text{-}0}}$
00011110100000001	{ (PTC-1, *en-1*), (SMO-0, *en-1*), (SMO-0, EN-1), (PTC-1, EN-1), (PTC-1, *ci-0*), (SMO-0, *ci-0*), (CI-0, SMO-0), (PTC-1, CI-0), (PTC-1, *hh-1*), (SMO-0, *hh-1*), (PTC-1, HH-1), (CIR-0, SMO-0), (HH-1, SMO-0) }	(nWG-1, PTC-1, SLP-0, nHH-0)	$\mathbf{M_7}$, $\mathbf{M_{12}}$
00000000100110100	{(CIR-1, SLP-0)}	(nWG-0, nHH-0, PTC-1, SLP-0)	$\mathbf{M_{14}}$,$\mathbf{M_{SLP\text{-}0}}$
10000000100110100	{ (nWG-0, SLP-1, *wg-0*), (nWG-0, nHH-0, SLP-1), (CIR-1, SLP-1, nWG-0), (SLP-1, nWG-0, CIA-0), (SLP-1, SMO-0, nWG-0), (SLP-1, nWG-0, WG-0), (SLP-1, nWG-0, PH-0), (SLP-1, *ptc-0*, nWG-0) }	(SLP-1, nWG-0, nHH-0, PTC-1)	$\mathbf{M_{13}}$,$\mathbf{M_{14}}$

Table 3: **Minimal observability and control sets for the *drosophila* single-cell SPN.** The attractors label the node states in order of SLP, *wg*, WG, *en*, EN, *hh*, HH, *ptc*, PTC, PH, SMO, *ci*, CI, CIA, CIR, nHH, nWG. The associated complex modules are colored to correspond to Table 1. Here, $\mathbf{M}_4$ indicates $\mathbf{M}_{nHH-0}$; $\mathbf{M}_6$ indicates $\mathbf{M}_{SLP-1,nHH-1}$; $\mathbf{M}_7$ indicates $\mathbf{M}_{SLP-0,nWG-1}$; $\mathbf{M}_9$ indicates $\mathbf{M}_{PTC-0}$, $\mathbf{M}_{10}$ indicates $\mathbf{M}_{SLP-0,nWG-1,nHH-1}$, $\mathbf{M}_{11}$ indicates $\mathbf{M}_{nWG-0,nHH-1}$, $\mathbf{M}_{12}$ indicates $\mathbf{M}_{nHH-0,PTC-1}$, $\mathbf{M}_{13}$ indicates $\mathbf{M}_{SLP-1,nHH-0,PTC-1}$; finally, $\mathbf{M}_{14}$ indicates $\mathbf{M}_{nWG-0,nHH-0,PTC-1}$.

Attractor	Expressed nodes	Minimal observability sets	Associated modules
Wild-type	(wg_4; WG_4; en_1; EN_1; hh_1;<br>HH_1; $ptc_{2,4}$; $PTC_{2,3,4}$; $ci_{2,3,4}$;<br>$CI_{2,3,4}$; $CIA_{2,4}$; CIR_3)	{ (CIR_3-1, PTC_1-0),<br>(PTC_1-0, ptc_2-1),<br>(CIA_2-1, PTC_1-0),<br>(PH_2-1, PTC_1-0),<br>(PTC_1-0, SMO_3-0) }	**M_7**/**M_9**,<br>**M_{11}**,<br>**M_{13}**<br>**M_6**
Broad stripes	($wg_{3,4}$; $WG_{3,4}$; $en_{1,2}$; $EN_{1,2}$;<br>$hh_{1,2}$; $HH_{1,2}$; $ptc_{3,4}$; $PTC_{3,4}$;<br>$ci_{3,4}$; $CI_{3,4}$; $CIA_{3,4}$)	{ (en_1-1, en_2-1),<br>(en_2-1, ptc_4-1),<br>(en_1-1, ptc_3-1),<br>(en_1-1, wg_3-1),<br>(wg_4-1, en_2-1) }	**M_{10}**,<br>**M_{10}**,<br>**M_6**,<br>**M_6**
No segmentation	($ci_{1,2,3,4}$; $CI_{1,2,3,4}$;<br>$PTC_{1,2,3,4}$; $CIR_{1,2,3,4}$)	{ (CIR_2-1, CIR_4-1),<br>(CIR_3-1, CIR_1-1),<br>(SLP_3-1, CIR_1-1),<br>(SLP_4-1, CIR_2-1),<br>(SLP_3-1, CIR_2-1),<br>(SLP_4-1, CIR_1-1) }	**M_{14}**<br>**M_{14}**<br>**M_{13}**<br>**M_{13}**
Wild-type variant	(wg_4; WG_4; en_1; EN_1; hh_1;<br>HH_1; $ptc_{2,4}$; $PTC_{1,2,3,4}$;<br>$ci_{2,3,4}$; $CI_{2,3,4}$; $CIA_{2,4}$; CIR_3)	{ (en_1-1, SMO_1-0),<br>(SMO_1-0, ptc_4-1),<br>(SMO_1-0, ptc_2-1),<br>(wg_4-1, SMO_1-0) }	**M_7**/**M_{12}**,<br>**M_{11}**,<br>**M_{13}**<br>**M_6**
Ectopic	(wg_3; WG_3; en_2; EN_2; hh_2;<br>HH_2; $ptc_{1,3}$; $PTC_{1,3,4}$; $ci_{1,3,4}$;<br>$CI_{1,3,4}$; $CIA_{1,3}$; CIR_4)	{ (CIR_4-1, PTC_2-0),<br>(ptc_1-1, PTC_2-0),<br>(CIA_1-1, PTC_2-0),<br>(PH_1-1, PTC_2-0),<br>(SMO_4-0, PTC_2-0) }	**M_{11}**,<br>**M_7**/**M_9**,<br>**M_6**,<br>**M_{13}**
Ectopic variant	(wg_3; WG_3; en_2; EN_2; hh_2;<br>HH_2; $ptc_{1,3}$; $PTC_{1,2,3,4}$; $ci_{1,3,4}$;<br>$CI_{1,3,4}$; $CIA_{1,3}$; CIR_4)	{ (SMO_2-0, en_2-1),<br>(SMO_2-0, ptc_3-1),<br>(SMO_2-0, wg_3-1),<br>(SMO_2-0, ptc_1-1) }	**M_{11}**,<br>**M_7**/**M_{12}**,<br>**M_6**,<br>**M_{13}**

Table 4: **Attractors and minimal observability sets for the *drosophila* parasegment SPN.** A node or s-unit's index indicates to which cell in the parasegment it belongs. In addition to the expressed nodes listed, every attractor has input nodes $SLP_{1,2}$ =OFF, $SLP_{3,4}$ =ON. The associated intracellular complex modules are listed for cells 1-4 (top to bottom) in the parasegment. Only the main complex modules for each cell are listed; they are colored to correspond to Table 1. Here, $\mathbf{M}_6$ indicates $\mathbf{M}_{SLP-1,nHH-1}$; $\mathbf{M}_7$ indicates $\mathbf{M}_{SLP-0,nWG-1}$; $\mathbf{M}_9$ indicates $\mathbf{M}_{PTC-0}$, $\mathbf{M}_{10}$ indicates $\mathbf{M}_{SLP-0,nWG-1,nHH-1}$, $\mathbf{M}_{11}$ indicates $\mathbf{M}_{nWG-0,nHH-1}$, $\mathbf{M}_{12}$ indicates $\mathbf{M}_{nHH-0,PTC-1}$, $\mathbf{M}_{13}$ indicates $\mathbf{M}_{SLP-1,nHH-0,PTC-1}$; finally, $\mathbf{M}_{14}$ indicates $\mathbf{M}_{nWG-0,nHH-0,PTC-1}$. Note that observability sets only require two sensors to reveal the given attractor.

	Nodes	S-units	Attractors	$\overline{D}(\Pi^*)$	$\lvert\Pi^*\rvert$	avg. $\lvert S_i\rvert \in \Pi^*$	max $\lvert S_i\rvert \in \Pi^*$
Example GRN	6	12	5	0.83	4	3.25	5 (83%)
Drosophila single-cell	17	34	10	0.81	8	5.25	16 (94%)
Thaliana	15	30	10	0.98	11	2.82	11 (73%)
Yeast	12	24	11	1.0	7	3.43	12 (100%)
Drosophila parasegment	60	120	?	0.87	32	4.44	33 (55%)
Leukemia	60	120	?	0.64	41	4.95	42 (70%)

Table 5: **Comparison of dynamical modularity between genetic regulatory networks.** The optimal cover Π^* is estimated for each network by maximizing the mean dynamical modularity among covers across seed set sizes, assuming pinning perturbation. Six networks are considered: the example GRN from Fig. 2 in the main text, the *drosophila* single-cell and parasegment SPNs [1], the *Thaliana arabadopsis* cell-fate specification network [6], the yeast cell-cycle network [7], and the T-LGL leukemia network [8]. For the first grouping of networks, Π^* was calculated considering all possible covers of $q \leqslant 6$ core complex modules with seed set size $s \leqslant 5$, with higher q values used when computationally feasible. By contrast, Π^* for the *drosophila* parasegment and leukemia networks was estimated using the greedy heuristic mentioned in Section 2.3. For each network the mean dynamical modularity of the optimal cover, $\overline{D}(\Pi^*)$, the size of the estimated optimal cover, $|\Pi^*|$, the average module size within the optimal cover, avg. $|S_i| \in \Pi^*$, and the maximum module size within the optimal cover, max $|S_i| \in \Pi^*$, is shown. Percentages are calculated based on the number of nodes in the network.

	Average driver set size	Min driver set size	Max driver set size	Driver superset size	FVS size
Example GRN	2	2	2	2	3
Drosophila single-cell	3.7	3	4	5	5
Thaliana	6.1	5	7	9	9
Yeast	6.45	5	7	7	8

Table 6: **Comparison of attractor controllability between genetic regulatory networks.** Statistics for the driver sets sufficient to fully resolve each fixed point attractor in the network, assuming pinning perturbation, are shown. Driver sets are equivalent to the seed sets of the minimal pathway modules whose unfolding includes all node states found in the respective attractor. The superset of driver nodes needed (based on the set of s-units sufficient to control to each attractor) is compared to that predicted by feedback vertex set theory [9, 10]. See the caption of Fig. 5 for a description of the networks used here.

	Yeast	*Thaliana*	*Drosophila* single-cell	*Drosophila* parasegment	Leukemia
Network size	12	15	17	60	60
Number of attractors	11	10	10	?	?
s = 1<br>number of modules,<br>maximum module size<br>(% of network)	17,<br>4 (33%)	16,<br>8 (53%)	14,<br>9 (53%)	40,<br>13 (22%)	48,<br>24 (40%)
s = 2<br>number of modules,<br>maximum module size<br>(% of network)	12,<br>10 (83%)	7,<br>12 (80%)	8,<br>16 (94%)	40,<br>28 (47%)	64,<br>41 (68%)
s = 3<br>number of modules,<br>maximum module size<br>(% of network)	12,<br>12 (100%)	3,<br>14 (93%)	6,<br>17 (100%)	152,<br>52 (87%)	223,<br>53 (88%)
s = 4<br>number of modules,<br>maximum module size<br>(% of network)	4,<br>12 (100%)	0,	0,	472,<br>56 (93%)	646,<br>54 (90%)
s = 5<br>number of modules,<br>maximum module size<br>(% of network)	2,<br>12 (100%)	0,	0,	1073,<br>59 (98%)	1446,<br>56 (93%)
s = 6<br>number of modules,<br>maximum module size<br>(% of network)	0,	0,	0,	1475,<br>60 (100%)	2353,<br>57 (95%)
Min fixed point seed set size (% of network)	3 (25%)	4 (27%)	3 (18%)	6 (10%)	8 (13%)

Table 7: **Comparison of core complex modules between genetic regulatory networks.** The number of core complex modules, along with the maximum module size, is found per network per seed set size s. Additionally, the minimal seed set size found to fully resolve every variable state (min fixed point seed set size) is shown; note that this fixed point is not necessarily an attractor of the original network, due to the pinning of the seeds involved. It is apparent that variable states in each network are quickly resolved as s is increased when considering the largest complex module. In comparing the larger networks, the leukemia network has more core complex modules than the *drosophila* parasegment per s value, suggesting more complicated dynamics. Percentages are calculated based on the number of variables (nodes) in the network. See the caption of Fig. 5 for a description of the networks used here.

REFERENCES

[1] Réka Albert and Hans G Othmer. The topology of the regulatory interactions predicts the expression pattern of the segment polarity genes in drosophila melanogaster. *Journal of theoretical biology*, 223(1):1–18, 2003.

[2] Manuel Marques-Pita and Luis M Rocha. Canalization and control in automata networks: body segmentation in drosophila melanogaster. *PloS one*, 8(3):e55946, 2013.

[3] Madalena Chaves, Eduardo D Sontag, and Réka Albert. Methods of robustness analysis for boolean models of gene control networks. *arXiv preprint q-bio/0605004*, 2006.

[4] Carlos Gershenson. Introduction to random boolean networks. *arXiv preprint nlin/0408006*, 2004.

[5] Rion Brattig Correia, Alexander J Gates, Xuan Wang, and Luis M Rocha. Cana: A python package for quantifying control and canalization in boolean networks. *arXiv preprint arXiv:1803.04774*, 2018.

[6] Alvaro Chaos, Max Aldana, Carlos Espinosa-Soto, Berenice García Ponce de León, Adriana Garay Arroyo, and Elena R Alvarez-Buylla. From genes to flower patterns and evolution: dynamic models of gene regulatory networks. *Journal of Plant Growth Regulation*, 25(4):278–289, 2006.

[7] Fangting Li, Tao Long, Ying Lu, Qi Ouyang, and Chao Tang. The yeast cell-cycle network is robustly designed. *Proceedings of the National Academy of Sciences*, 101(14):4781–4786, 2004.

[8] Ranran Zhang, Mithun Vinod Shah, Jun Yang, Susan B Nyland, Xin Liu, Jong K Yun, Réka Albert, and Thomas P Loughran. Network model of survival signaling in large granular lymphocyte leukemia. *Proceedings of the National Academy of Sciences*, 2008.

[9] Bernold Fiedler, Atsushi Mochizuki, Gen Kurosawa, and Daisuke Saito. Dynamics and control at feedback vertex sets. i: Informative and determining nodes in regulatory networks. *Journal of Dynamics and Differential Equations*, 25(3):563–604, 2013.

[10] Atsushi Mochizuki, Bernold Fiedler, Gen Kurosawa, and Daisuke Saito. Dynamics and control at feedback vertex sets. ii: A faithful monitor to determine the diversity of molecular activities in regulatory networks. *Journal of theoretical biology*, 335:130–146, 2013.
